# Bio-psychosocial factors of children and adolescents with internet gaming disorder: a systematic review

**DOI:** 10.1186/s13030-019-0144-5

**Published:** 2019-02-14

**Authors:** Nagisa Sugaya, Tomohiro Shirasaka, Kenzo Takahashi, Hideyuki Kanda

**Affiliations:** 1 Unit of Public Health and Preventive Medicine, School of Medicine, Yokohama City University, 3-9 Fukuura, Kanazawa-ku, Yokohama, 236-0004 Japan; 2 Department of Psychiatry, Teine Keijinkai Hospital, 1-12-1-40 Maeda, Teine-ku, Sapporo, Hokkaido 006-8555 Japan; 3 Teikyo University Graduate School of Public Health, 2-11-1 Kaga, Itabashi-ku, Tokyo, 173-8605 Japan; 4 Faculty of Medicine, Department of Environmental Medicine and Public Health, Shimane University, 89-1 Enya-cho, Izumo-shi, Shimane 693-8501 Japan

**Keywords:** Internet gaming disorder, Children, Adolescents

## Abstract

Previous large-scale studies suggest that internet gaming disorder (IGD) among children and adolescents has become an important public concern. Minors are known to be particularly susceptible to problematic internet gaming use owing to age-related underdevelopment of cognitive control. It has been shown that precursors of addictions appear during adolescence; therefore, prevention efforts must be established targeting minors who have their first experience with addictive substances and behaviors during pubescence. Since the DSM-5 classification of IGD in 2013, studies on IGD have drastically increased in number. Thus, we performed an updated review of studies of IGD in children and adolescents to assess the clinical implications of IGD. The search included all publication years, using PubMed, MEDLINE, and PsycINFO. Across studies, the presence of IGD had a negative effect on sleep and schoolwork in minors. Additionally, family factors, including the quality of parent-child relationships, were important social factors in minors with IGD. Brain imaging studies indicate that impaired cognitive control in minors with IGD is associated with abnormal function in the prefrontal cortex and striatum. Persistent pathological online game use from childhood may aggravate abnormal brain function; therefore, preventive care and early intervention are increasingly important. Although extant research supports the effectiveness of cognitive behavioral therapy for minors with IGD, effective psychological intervention for minors with IGD is an urgent issue that requires further research. This review, which presents updated findings of IGD in minors, is expected to contribute to the development of future research and be useful in clinical practice in the field of child and adolescent psychiatry.

## Background

Internet gaming disorder (IGD) was recently included as a tentative disorder in the latest (fifth) edition of the American Psychiatric Association’s (APA) Diagnostic and Statistical Manual of Mental Disorders (DSM-5) [1]. The DSM-5 summarizes IGD as the “persistent and recurrent use of the Internet to engage in games, often with other players, leading to clinically significant impairment or distress,” as indicated by five or more out of nine proposed items (Table 1). Gaming disorder is under consideration for inclusion as a new diagnostic category in the WHO ICD-11 (International Classification of Diseases, 11th revision). Before the DSM-5 classification for IGD, previous studies employed various questionnaires to classify IGD or problematic online game play. However, the criteria used were not completely consistent. The Beard Diagnostic Questionnaire [2] does not assess criterion E of the DSM-5 IGD classification. Similarly, Young’s Internet Addiction Test (IAT) [3] and the Young Diagnostic Questionnaire (YDQ) [4] do not assess criterion F [5]. The Scale for the Assessment of Internet and Computer game Addiction—Gaming Module (AICA-S-gaming) [6] does not include criterion E, G, or I of the DSM-5 classification. Thus, the classification of IGD in the DSM-5 has played an important role in the accumulation of reliable study results for IGD.

**Table 1:** Diagnostic criteria of IGD in DSM-5

Repetitive use of internet-based games, often with other players, that leads to significant issues with functioning. Five of the following criteria must be met within 1 year:	
A. Preoccupation or obsession with internet games.	
B. Withdrawal symptoms when not playing internet games.	
C. A build-up of tolerance – more time needs to be spent playing the games.	
D. The person has tried to stop or curb playing internet games, but has failed to do so.	
E. The person has had a loss of interest in other life activities, such as hobbies.	
F. The person has had continued overuse of internet games even with the knowledge of how much they impact a person’s life.	
G. The person lied to others about his or her internet game usage.	
H. The person uses internet games to relieve anxiety or guilt – it is a way to escape.	
I. The person has lost or put at risk an opportunity or relationship because of internet games.	

According to the latest survey by the Entertainment Software Association (ESA), which gathered data from more than 4000 households in the U.S., 65% of households were home to at least one person who played computer and video games regularly, and 29% of the players were under the age of 18 years [7]. Previous large-scale studies suggest that IGD among minors has become an important public concern in many areas, e.g., Asia [8, 9], Australia [5, 10], and Europe [11–15]. Minors are known to be particularly susceptible to problematic use of internet gaming [16, 17] because of their immature cognitive control during this period [18–20]. In addition, one study compared online and offline gamers and indicated that online gamers were more likely to score higher on overuse, interpersonal conflict, and social isolation subscales of the Problematic Online Gaming Questionnaire (POGQ) [21]. Prevention efforts must be established that target adolescents who have their first experiences with addictive substances and behaviors during pubescence [17] because it appears that addictions tend to have precursors that appear during adolescence [22]. Additionally, children and adolescents are particularly expected to exhibit prominent relationships among the family environment, parent-child relationship, academic performance, and IGD symptoms; thus, the pathophysiology of IGD in children and adolescents should be understood from a different perspective to that of adults.

A review of IGD in children and adolescents was published by Kuss et al. [17] in 2012. Since the DSM-5 classification of IGD was published in 2013, the number of articles about IGD has drastically increased. Thus, we reviewed previous studies of IGD in children and adolescents to gain a better understanding of biopsychosocial factors of IGD.

## Methods

The literature search utilized a systematic and structured approach adopting the PRISMA guidelines for systematic reviews and meta-analyses [23]. The search included all publication years (up to February 2018) using the major medical and psychological literature databases, including PubMed, MEDLINE, and PsycINFO. The title or abstract keywords used for the systematic search were: (“internet” or “online” or “computer”) AND (“game” or “gaming”) AND (“addiction” or “addictive” or “disorder” or “problematic” or “pathological” or “excessive” or “overuse” or “abuse” or “compulsive”) AND (“child” or “teenager” or “adolescent” or “minor”). Furthermore, the reference lists of selected articles were also examined for additional suitable publications. With respect to PsycINFO, we narrowed the search to research articles written in English and including participants aged 28 years or younger because the search refinement option for age of PsycINFO allows selecting only samples aged “0 to 18” and “18 to 28.”

The inclusion criteria upon which the studies were selected were as follows: (1) written in English, (2) the inclusion of minors younger than 20 years in the sample because The World Health Organization defines an adolescent as any person aged 10–19 years, (3) publication as an original paper but not as a review or case report, (4) full text availability, and (5) investigation of biopsychosocial characteristics in minors with IGD. The purpose of our review is to summarize the multi-dimensional characteristics of children and adolescents with IGD; thus, we excluded the studies that evaluated only the degree of problematic internet game use in the general population.

**Fig. 1:**
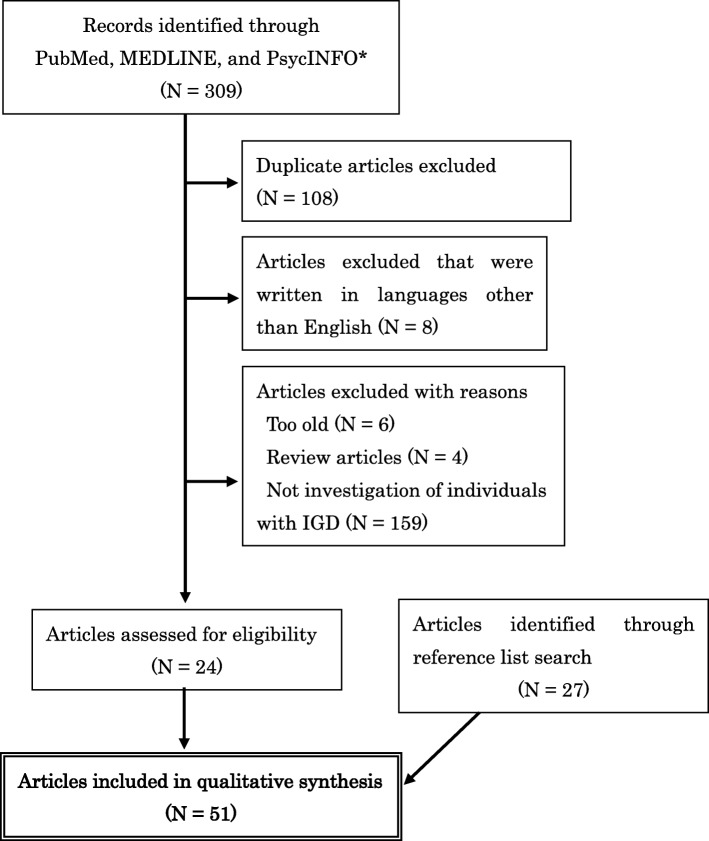
Summary of the search and inclusion process. * Regarding PsycINFO, the database search was narrowed to include only research articles written in English and including participants aged 28 or younger

Figure 1 presents a summary of the search and inclusion process for this review. The database search in PubMed identified 98 manuscripts. Sixty-seven manuscripts were identified from MEDLINE. The database search in PsycINFO identified 144 manuscripts. One hundred and eight duplicate manuscripts among the three databases were excluded. The evaluation of titles and abstracts resulted in the exclusion of 177 studies as they did not meet the inclusion criteria (8 manuscripts written in languages other than English; 6 manuscripts of studies involving participants older than 20 years; 4 review articles; 159 manuscripts of studies that did not involve individuals with IGD). Twenty-seven studies were identified from the reference lists of those identified articles. We identified 51 potential articles for inclusion in this review.

Authors, experienced in IGD studies, selected articles that met the inclusion criteria and confirmed the study content. Subsequently, we classified the content of each article into the following categories: prevalence and classification of IGD, profile of IGD symptoms, psycho-social features, psychological intervention, and biological features.

In this review, the term “IGD” is used when we refer to the results of the studies using the DSM-5 classification. Regarding the studies that used other methods of classification, we quote the name of the disorder used in each study (e.g., problematic Internet game user, pathological computer game player).

## Results

### Prevalence and classification of IGD

Table 2 shows the prevalence of IGD from previous epidemiological studies. The results of studies employing the DSM-5 criteria indicated an incidence of IGD of 1.2–5.9% in teenagers [5, 13, 14, 24, 25]. Those previous studies indicated that the proportion of boys with IGD was higher than that of girls.

**Table 2:** Prevalence of IGD

Author (year)	N of participants	Prevalence of IGD			Diagnostic criteria	Mean age of participants	Country
		Total	Boys	Girls			
Wartberg et al. (2017) [24]	1095	2.4%	–	–	DSM-5	13.0 (SD = 0.8)	Germany
Yu et al. (2016) [25]	2024	5.9%	10.4%	1.2%	DSM-5	14.5 (SD = 0.5)	South Korea
King et al. (2016) [5]	824	3.1%	5.7%	0.7%	DSM-5	14.1~14.5 (SD = 1.2~1.5)	Australia
Pontes et al. (2016) [13]	1071	2.4%	–	–	DSM-5	13.4 (SD = 0.6)	Slovenia
Rehbein et al. (2015) [14]	11,003	1.2%	2.0%	0.3%	DSM-5	14.9 (range = 13–18)	Germany
Johansson et al. (2004) [26]	3237	2.7%	4.2%	1.1%	Young Diagnostic Questionnaire	12~18	Norway
Strittmatter et al. (2015) [15]	8807	3.6%	–	–	Young Diagnostic Questionnaire	15.0 (SD = 1.3)	Estonia, Germany, Italy, Romania and Spain
Müller et al. (2015) [12]	112,938	1.6%	3.1%	0.3%	Assessment of Internet and Computer game Addiction -Gaming Module	15.8 (SD = 0.7)	Germany, Greece, Iceland, the Netherlands, Poland, Romania, and Spain
Vadlin et al. (2015) [30]	1814	1.3% *2.4%	–	–	Gaming Addiction Identification Test and its parent version	13~15	Sweden
Kiraly et al. (2014) [28]	4875	4.3%	–	–	12-item Problematic Online Gaming Questionnaire Short-Form	16.4 (SD = 0.9)	Hungary
Pápay et al. (2013) [27]	5045	4.6%	–	–	12-item Problematic Online Gaming Questionnaire Short-Form	16.4 (SD = 0.9)	Hungary
Van Rooij et al. (2011) [29]	1572	3.6%	–	–	Compulsive Internet Use Scale	14.4 (SD = 1.2)	the Netherlands

* rated by adolescents’ parents

The studies using the YDQ [4] reported that 3.6% of adolescents have problematic internet game use [15] and that 2.7% of adolescents are pathological computer game players [26]. Müller et al. [12] indicated that 1.6% of adolescents have IGD, as assessed using the AICA-S-gaming [6] for classification. Pápay et al. [27] and Király et al. [28] conducted a study employing the 12-item POGQ Short-Form and reported problematic online game use in adolescents of 4.6 and 4.3%, respectively. Van Rooij et al. [29] reported that 3.6% of adolescents have an online gaming addiction, as assessed using the Compulsive Internet Use Scale.

Vadlin et al. [30] reported the prevalence of IGD as rated by adolescents and their parents. The difference between the prevalence of IGD in adolescents based on self-rating (1.3%) and that by parents (2.4%) was significant.

As detailed above, depending on the criteria used, about one in every 20–80 minors seems to meet the criteria for IGD.

Meanwhile, a recent report on 7865 adolescent European gamers by Colder Carras et al. [31] promoted the awareness of inappropriate classification by using traditional approaches to IGD classification, such as scale score cut-offs. The “engaged gamer group,” who had moderate symptom levels and moderate to low problem levels classified based on their report of gaming-related symptoms/problems, showed that those with average AICA-S scores were classified as being at risk for IGD and had poor psychosocial well-being.

### Profile of IGD symptoms

The most commonly reported symptoms among individuals with IGD were escape (96%), preoccupation (92%), tolerance (77%), and continued use despite harm (77%) [5].

Some studies reported that gaming time in adolescents with IGD was about 3–6 h (King et al. [5]: 27.8 ± 1.3 h/week [i.e., 3.96 h/day]; Kim et al. [32]: 227.29 ± 145.49 min/day [i.e., 3.79 ± 2.42 h/day]; Dreier et al. [11]: 6.15 ± 3.51 h/day; Rehbein et al. [14]: 375.36 ± 373.93 min/day [6.26 ± 6.23 h/day]).

Regarding game genre, adolescents with IGD were reported to predominantly play shooting action games (96%) and massive multiplayer online games (81%) compared with at-risk and non-problem cases [5]. Additionally, the severity of IGD was reported to be predicted by the preferred genre of online game (single player game, massive multiplayer online role-playing games [MMORPGs], first-person shooters [FPS], and strategy games), and the use of MMORPGs and shooter games were the strongest predictors [12].

The presence of IGD was reported to interfere with daily living, including schoolwork. The number of school classes missed in the past 6 months and any gaming-related truancy in the past 6 months were more frequent in secondary school students with IGD (19.34 ± 43.44 times and 65.63 ± 47.68%, respectively) than in the non-IGD group (4.70 ± 17.84 times and 3.92 ± 19.40%, respectively), and the grade point average of students with IGD was worse than that of those without IGD [14]. Another study also reported that secondary school students with IGD had lower academic performance than non-problematic gamers and non-gamers [12]. Secondary school students with IGD showed decreased activity compared to non-problematic gamers and non-gamers [12]. The sleep disturbance score of secondary school students with IGD was higher than that of a non-IGD group [14].

### Psycho-social features

#### Various psychological and social problems

Various psychological and social problems were found in adolescents with IGD. A problematic internet game use group showed more severe depression, conduct disorder, emotional symptoms, hyperactivity, peer problems, and perceived stress, and a higher percentage of self-injurious behavior compared with a normal internet use group [15]. Another study reported that secondary school students with IGD exhibited more severe rule-breaking behavior, aggressive behavior, somatic complaints, and emotional, social, and cognitive problems compared to at-risk gamers, non-problematic gamers, and non-gamers [12]. King et al. [5] reported higher scores of depression, anxiety, and stress in adolescents with IGD. Dreier et al. [11] reported that adolescents with IGD showed higher stress levels and severe psychosocial problems, including problems with peers, hyperactivity, concentration difficulties, applied dysfunctional coping strategies more frequently, and spent a larger amount of money on free-to-play browser games compared with non-problematic gamers.

#### Impulsiveness

Impulsiveness has received attention as an important psychological characteristic of adolescents with IGD. Brain imaging studies have employed the Barratt impulsiveness scale-11 (BIS) [33] to assess impulsiveness in adolescents with IGD. These studies found higher impulsiveness in the IGD group in comparison with the healthy control group [34–37]. Regarding the association between impulsiveness and brain imaging, self-rated impulsiveness was positively associated with activation of the left superior medial frontal gyrus [34] and topological alterations over the frontolimbic connections [38] in adolescents with IGD, while other studies reported no significant correlation between impulsiveness and cerebral blood flow values [36] or voxel-mirrored homotopic connectivity [37].

#### Comorbidity with ADHD

Regarding impulsiveness, the relationship between problematic online game use and attention-deficit/hyperactivity disorder (ADHD) has also attracted research attention. In adolescents, a study of patients with ADHD and problematic online game play who were treated with methylphenidate or atomoxetine for 3 months showed that improvement in the severity of internet addiction was positively correlated with a reduction in impulsivity [39].

#### Sensation seeking

Based on the biosocial-affect model of adolescent problem behavior [40] and the Dual Systems Model [41, 42], Hu et al. [43] investigated the mechanisms underlying the relationship between sensation seeking and IGD in male adolescents at risk for IGD. The results showed that sensation seeking, positive affective associations with online games, and impulsivity were each significantly and positively associated with online gaming addiction in adolescents. Positive affective associations mediated the relationship between sensation seeking and online gaming addiction. Furthermore, impulsivity moderated the relationship between positive affective associations and online gaming addiction, such that the association between positive affective association and online gaming addiction was stronger for adolescents with high than low impulsivity.

#### Cognitive factors related to IGD

King et al. [5] found that adolescents with IGD may have specific maladaptive beliefs that differentiate them from other gaming populations, including adolescents without IGD who are highly engaged in video gaming. These cognitions were categorized as follows: (1) the overvaluation of gaming rewards and identities, (2) inflexible rules and biases that arise in gaming situations, (3) over-reliance on gaming to meet self-esteem needs, and (4) gaming as a method of gaining social acceptance. The results of this study indicated a linear positive relationship between the presence of maladaptive gaming cognitions, particularly maladaptive rules about gaming and gaming-based self-esteem, and IGD symptomatology.

#### Family factors

Family environment plays an important role in psychiatric disorders, including IGD, particularly in children and adolescents. Bonnaire et al. [44] investigated the relationships between parental attitudes, adolescent perception of family function, and IGD. Their results showed that non-problematic gamers have better family cohesion, while problematic gamers have more family conflict and a poorer relationship with their family. Rules about gaming use are important in male adolescents to prevent IGD, while for female adolescents, banning them from gaming could prevent them from excessive gaming use. For both sexes, parental monitoring, conflicts, and family relationship were associated with IGD. Kim et al. [45] also reported that internet gaming addiction is associated with parental attachment and attitude toward parenting. Wartberg et al. [46] observed significant associations between the presence of IGD in early adolescence and parental anxiety, in addition to factors related to adolescents, including male sex, a higher degree of adolescent anti-social behavior, anger control problems, emotional distress, self-esteem problems, and hyperactivity/inattention. However, a previous study did not find a strong association between child-parent relationship and IGD [47].

#### Interview studies

Using a semi-structured interview at internet cafes, Wong et al. [48] identified that 5/13 adolescents (38.5%) had a pathological gaming problem and 2/13 adolescents were problem gamers (15.4%). The psychological factors associated with gaming addiction were identified as low self-esteem, a strong desire for aggressive and exciting experiences, reliance on gaming to kill time and to obtain satisfaction, coping with problems and negative emotions, and obsession with achieving higher rankings in games. The social and environmental risk factors were accessibility to internet cafes, aggressive promotional activities at internet cafes, peer pressure, family influence and early gaming experiences, perceived parental approval, lack of parental supervision, and poor family relationships.

Another interview study using content analysis [49] reported five categories with distinct themes: (1) addicts’ psychological needs and motivations, (2) online games as the everyday focus of the addicts, (3) the interplay between the “real” self and “virtual” self, (4) online games as a compensatory or extensive satisfaction for addicts’ needs, and (5) addicts’ self-reflections.

### Psychological interventions

A brief 3-week family therapy course for adolescents with online gaming addiction improved perceived family cohesion and online game addiction symptoms [50]. Sakuma et al. [51] demonstrated the effectiveness of a nine day “self-discovery camp” as a Japanese version of a therapeutic residential camp for adolescents with IGD on their gaming time and self-efficacy. In this previous study, adolescents with IGD showed decreased gaming time and increased self-efficacy as well as the extent to which they take action toward ceasing their addictive behavior 3 months after the camp. Those who participated in the camp were not allowed to bring personal computers, mobile phones, gaming machines, or any other digital equipment, and they experienced cognitive behavioral therapy sessions, medical lectures, personal counseling sessions, a workshop about relationships with the internet, and outdoor activities.

### Biological features

#### Resting state brain imaging studies of IGD

The results of resting state brain imaging studies are shown in Table 3. Regarding brain volume, minors with IGD showed decreased gray matter volume in prefrontal cortex (PFC) regions, including the bilateral dorsolateral prefrontal cortex (DLPFC), orbitofrontal cortex (OFC), anterior cingulate cortex (ACC), and the right supplementary motor area (SMA) in comparison with healthy controls [52]. In contrast, another study reported no intergroup differences in gray matter volume within the right dorsomedial prefrontal cortex (dmPFC), bilateral insula, OFC, right amygdala, and left fusiform cortex [35]. Wang et al. [53] reported decreased gray matter volume in the bilateral ACC, precuneus, SMA, superior parietal cortex, left DLPFC, left insula, and bilateral cerebellum in the IGD group.

**Table 3:** Characteristics of resting state brain imaging data in individuals with IGD

Author (year)	Participants	Diagnostic criteria	Brain imaging methods	Results
Han et al. (2017) [54]	IGD group: *N* = 78 (boys), 14.7 ± 2.0 years. Control group: *N* = 73 (boys), 14.6 ± 1.8 years.	1) Excessive online game play time (more than 4 h per day/30 h per week); 2) IAT scores > 50; 3) Irritable, anxious and aggressive behavior when forced to stop online game play; 4) Impaired behaviors or distress, economic crisis and maladaptive regular life patterns including disrupted diurnal rhythms, irregular meals, failure to maintain personal hygiene and school refusal.	Method: fMRI (functional connectivity) Scanner: 3 T Software: AFNI, SPM12b, MatLab	IGD group showed increased functional connectivity between seven pairs of regions; left frontal eye field to dorsal anterior cingulate, left frontal eye field to right anterior insula, left DLPFC to left TPJ, right DLPFC to right TPJ, right auditory cortex to right motor cortex, right auditory cortex to supplementary motor area, and right auditory cortex to dorsal anterior cingulate.
Park et al. (2017) [38]	IGA group: N = 19 (boys) 13.6 ± 1.0 years. Control group: *N* = 20 (boys), 13.4 ± 0.9 years.	Korean Internet Addiction Proneness Scale	Method: fMRI Scanner: 3 T Software: SPM8 and Data Processing Assistant for Resting-State Functional (DPARSF)	1) Brain functional networks in IGA group showed higher global efficiency and lower local efficiency relative to the controls; IGA induced brain functional networks to shift toward a random topological architecture. 2) IGA group exhibited consistently higher regional global efficiency over the frontal-sensorimotor, frontal-temporal, frontal-limbic, and temporal region, and lower regional local efficiency in the sensorimotor and limbic regions than those of the controls. 3) Severe impulsiveness in IGA group was associated with topological alterations over frontolimbic connections.
Jin et al. (2016) [52]	IGD group: *N* = 25 (16 boys and 9 girls), 19.1 ± 1.1 years. Control group: *N* = 21 (14 boys and 7 girls), 18.8 ± 1.8 years.	DSM-5	Method: VBM and fMRI (functional connectivity) Scanner: 3 T Software: FMRIB Software Library (FSL) 4.1	IGD group showed: 1) Decreased gray matter volume in PFC regions including the bilateral DLPFC, OFC, ACC, and the right SMA. 2) Decreased functional connectivity between several cortical regions and authors’ seeds, including the insula, and temporal and occipital cortices. 3) Decreased functional connectivity between some subcortical regions, i.e., dorsal striatum, pallidum, and thalamus.
Du et al. (2016) [35]	IGD group: *N* = 25 (boys), 17.3 ± 3.4 years. Control group: *N* = 27 (boys), 17.5 ± 2.9 years.	[1] five or more “yes” responses on the YDQ for internet addition; [2] online game playing time ≥ 4 h per day; and [3] IAT score ≥ 50.	Method: VBM Scanner: 3 T Software: VBM8 toolbox of the Statistical Parametric Mapping (SPM) 8	Region-of-interest analysis revealed that gray matter volume in the right dmPFC, bilateral insula and OFC, right amygdala, and left fusiform gyrus showed significant positive correlations with impulsivity in the control group, while no significant correlation was found in the IGD group.
Wang et al. (2015) [37]	IGD group: N = 17 (13 boys and 4 girls), 16.9 ± 2.7 years. Control group: *N* = 24 (18 boys and 6 girls), 15.9 ± 2.7 years.	Modified YDQ for internet addiction criteria (answered “yes” to questions 1 through 5 and at least any one of the remaining three questions).	Method: fMRI Scanner: 3 T Software: DPARSF3.0 Advanced edition MRImaging toolkit	IGD group showed decreased voxel-mirrored homotopic connectivity (VMHC) between the left and right superior frontal gyrus (orbital part), inferior frontal gyrus (orbital part), middle frontal gyrus, and superior frontal gyrus.
Wang et al. (2015) [53]	IGD group: N = 28 (18 boys and 10 girls), 18.8 (1.33) years. Control group: *N* = 28 (20 boys and 8 girls), 19.3 (2.56) years.	Modified YDQ for internet addiction criteria	Method: VBM Scanner: 3 T Software: FSL-VBM	IGD group showed: 1) Decreased gray matter volume of the bilateral ACC, precuneus, SMA, superior parietal cortex, left DLPFC, left insula, and bilateral cerebellum. 2) Negative correlation between gray matter volume of the ACC and the incongruent response errors of Stroop task.
Hong et al. (2015) [96]	IGD group: *N* = 12 (boys), 13.41 ± 2.31 years. Control group: *N* = 11 (boys), 14.81 ± 0.87 years.	IAT score ≥ 50	Method: fMRI Scanner: 3 T Software: SPM8	IGD group showed: 1) Reduced dorsal putamen functional connectivity with the posterior insula-parietal operculum. 2) More time spent playing online games predicted significantly greater functional connectivity between the dorsal putamen and bilateral primary somatosensory cortices. 3) Significant and specific differences in the dorsal putamen functional connectivity.
Xing et al. (2014) [97]	IGD group: *N* = 17 (10 boys and 7 girls), 19.1 ± 0.7 years. Control group: N = 17 (11 boys and 6 girls), 19.8 ± 1.3 years.	IAT score ≥ 50	Method: fMRI (functional connectivity) and diffusion tensor imaging (DTI) tractography methods Scanner: 3 T Software: FMRIB’s Software Library (FSL)	IGD group showed: 1) Decreased fractional anisotropy (FA) in the right salience network (SN) tract and no significant differences in functional connectivity compared with the control group. 2) Negative correlation between FA values of the right SN tract and errors during the incongruent condition in a color-word Stroop task (i.e., impaired cognitive control).
Feng et al. (2013) [36]	IGA group: *N* = 15 (13 boys and 2 girls), 16.93 ± 2.34 years. Control group: *N* = 18 (14 boys and 4 girls), 16.33 ± 2.61 years.	Modified YDQ for internet addiction criteria	Method: fMRI Scanner: 3 T Software: SPM8	IGA group showed: 1) Higher global CBF in the left inferior temporal lobe/fusiform gyrus, left parahippocampal gyrus/amygdala, right medial frontal lobe/ACC, left insula, right insula, right middle temporal gyrus, right precentral gyrus, left SMA, left cingulate gyrus, and right inferior parietal lobe. 2) Lower CBF in the left middle temporal gyrus, left middle occipital gyrus, and right cingulate gyrus.
Ding et al. (2013) [98]	IGA group: *N* = 17 (13 boys and 4 girls), 16.94 ± 2.73 years. Control group: *N* = 24 (16 boys and 8 girls), 15.87 ± 2.69 years.	Modified YDQ for internet addiction criteria	Method: fMRI Scanner: 3 T Software: MRIcroN toolset, SPM5, and the Resting-State fMRI Data Analysis Toolkit	IGA group showed: 1) Increased functional connectivity in the bilateral cerebellum posterior lobe and middle temporal gyrus. 2) Decreased connectivity in the bilateral inferior parietal lobule and right inferior temporal gyrus. 3) Positive correlation between severity of internet addiction and connectivity in the PCC and right precuneus, posterior cingulate gyrus, thalamus, caudate nucleus, NAc, SMA, and lingual gyrus. 4) Negative correlation between severity of internet addiction and connectivity in the PCC and two areas (right cerebellum anterior lobe and left superior parietal lobule).

*ACC* anterior cingulate cortex

*CBF* cerebral blood flow

*DLPFC* dorsolateral prefrontal cortex

*dmPFC* dorsomedial prefrontal cortex

*NAc* nucleus accumbens

*OFC* orbitofrontal cortex

*PCC* posterior cingulate cortex

*PFC* prefrontal cortex

*SMA* supplementary motor area

*TPJ* temporoparietal junction

*VBM* voxel-based morphometric

*IAT* Internet Addiction Test

*YDQ* Young Diagnostic Questionnaire

*IGD* Internet Gaming Disorder

*OGA* Online Gaming Addiction

Regarding functional connectivity, an IGD group showed decreased functional connectivity between several cortical regions and authors’ seeds, including the insula and temporal and occipital cortices, and between some subcortical regions, i.e., the dorsal striatum, pallidum, and the thalamus [52]. Another study assessed the voxel-mirrored homotopic connectivity (VMHC) and reported decreased connectivity between the left and right superior frontal gyrus (orbital part), inferior frontal gyrus (orbital part), middle frontal gyrus, and the superior frontal gyrus in the IGD group [37]. Han et al. [54] reported that adolescents with IGD show increased functional connectivity between seven pairs of regions: the left frontal eye field to the dorsal anterior cingulate, the left frontal eye field to the right anterior insula, the left DLPFC to the left temporoparietal junction (TPJ), the right DLPFC to the right TPJ, the right auditory cortex to the right motor cortex, the right auditory cortex to the SMA, and the right auditory cortex to the dorsal anterior cingulate.

A study of the relationship between brain imaging data and impulsivity [35] revealed that the gray matter volume in the right dmPFC, bilateral insula, OFC, right amygdala, and left fusiform gyrus showed significant positive correlations with impulsivity in the control group but not in an IGD group. Another study reported a negative correlation between the gray matter volume of the ACC and incongruent response errors on the Stroop task, implying decreased cognitive control ability in the IGD group [53]. Dysfunctional frontolimbic connections have also been associated with more severe impulsiveness in those with IGD [38].

#### Task-related brain imaging studies of IGD

Table 4 shows the results of brain imaging studies for all tasks. The relationship between IGD and brain imaging in adolescents has been demonstrated using a task assessing cognitive control. A study using the Stroop test reported that adolescents with IGD show increased caudate nucleus and nucleus accumbens (NAc) volume, and that there are correlations between caudate nucleus volume and Stroop task performance and between NAc volume and the severity of internet addiction. This study demonstrated the important role of the striatum in the pathophysiology of IGD [55]. A study using the Go/No-Go task reported hyperactivity in the left superior medial frontal gyrus, right ACC, right superior/middle frontal gyrus, left inferior parietal lobule, left precentral gyrus, and left precuneus and cuneus, and hypoactivity in the bilateral middle temporal gyrus, bilateral inferior temporal gyrus, and right superior parietal lobule in adolescents with IGD during No-Go trials [34]. The participants with IGD in Ding et al. [34] showed a positive association between activation of the left superior medial frontal gyrus and self-rated impulsiveness and addiction severity.

**Table 4:** Characteristics of task-related brain imaging data in individuals with IGD

Author (year)	Participants	Diagnostic criteria	Brain imaging methods	Results
Cai et al. (2016) [55]	IGD group: N = 27 (23 boys and 4 girls), 17.1 ± 0.9 years. Control group: *N* = 30 (22 boys and 8 girls), 18.3 ± 1.3 years.	DSM-5	Method: MRI Scanner: 3 T Software: FreeSurfer v5.1.0 Task: Stroop test	IGD group showed: 1) Increased volumes of caudate nucleus and NAc. 2) Correlation between caudate nucleus volume and Stroop task performance (cognitive control). 3) Correlation between NAc volume and severity of internet addiction.
Chun et al. (2015) [58]	IGD group: *N* = 16 (boys), 13.63 ± 1.03 years. Control group: *N* = 19 (boys), 13.37 ± 0.90 years.	Korean Internet Addiction Proneness Scale	Method: fMRI Scanner: 3 T Software: SPM8 Task: Discriminating the level of the negative feeling induced by the word stimuli including swear words, affective words, and neutral words	IGD group showed: 1) Reduced activation in the right OFC related to cognitive control and in the dorsal ACC related to social rejection during the swear word condition. 2) Negative correlation between activity in the right amygdala toward swear words and anger control score.
Qi et al. (2015) [56]	IGD group: *N* = 23 (boys), 17.26 ± 3.56 years. Control group: *N* = 24 (boys), 17.42 ± 3.05 years.	[1] five or more “yes” responses on the YDQ for internet addition [2] IAT score ≥ 50	Method: fMRI Scanner: 3 T Software: SPM8 Task: the balloon analog risk task (BART) ※The BART evaluates the modulation of the risk level (the probability of balloon explosion)	IGD group showed: 1) Reduced modulation of activation of the right DLPFC in response to risk level during the active BART. 2) Negative correlation between risk-related DLPFC activation during the active BART and (self-rating) impulsivity.
Ding et al. (2014) [34]	IGD group: *N* = 17 (14 boys and 3 girls), 16.41 ± 3.20 years. Control group: N = 17 (14 boys and 3 girls), 16.29 ± 2.95 years.	Modified YDQ for internet addiction criteria	Method: fMRI Scanner: 3 T Software: SPM8 Task: Go/No-Go Task	IGD group showed: 1) Hyperactivity during No-Go trials in the left superior medial frontal gyrus, right anterior cingulate cortex, right superior/middle frontal gyrus, left inferior parietal lobule, left precentral gyrus, and left precuneus and cuneus. 2) Hypoactivity during No-Go trials in the bilateral middle temporal gyrus, bilateral inferior temporal gyrus, and right superior parietal lobule. 3) Positive association between activation of the left superior medial frontal gyrus and self-rated impulsiveness and addiction severity.
Kim et al. (2012) [57]	Excessive online game playing group: *N* = 13, 14.5 ± 1.1 years. Control group: *N* = 10, 14.2 ± 1.3 years.	1) IAT (≥50) 2) difficulty with daily life resulting from excessive game play	Method: fMRI Scanner: 3 T Software: The Brain Voyager software package Task: working memory task	Excessive online game playing group showed: 1) Greater activity in the right middle occipital gyrus, left cerebellum posterior lobe, left premotor cortex, and left middle temporal gyrus in response to working memory tasks during baseline measurements. 2) Increased activity within the right dorsolateral prefrontal cortex and left occipital fusiform gyrus after 4 weeks of treatment 3) Changes in the severity of online game playing were negatively correlated with changes in the mean beta value of the right dorsolateral prefrontal cortex in response to complex stimulation after 4 weeks of treatment.
Han et al. (2012) [50]	OGA: *N* = 15, 14.2 ± 1.5 years. Adolescents without problematic online game play: N = 15, 14.0 ± 1.3 years. (Over 3 weeks, families were asked to carry out homework assignments focused on increasing family cohesion for more than 1 h/day and 4 days/week)	1) Game playing time greater than 4 h per day and 30 h per week; 2) IAT ≥50; 3) impaired behaviors or distress due to excessive on-line game play which are modified from DSM-IV criteria for substance abuse	Method: fMRI Scanner: 3 T Software: The Brain Voyager software package Task: viewing affection and game scenes without responding	OGA group showed: 1) Decreased activity within the caudate, middle temporal gyrus, and occipital lobe in response to images depicting parental affection and increased activity of the middle frontal and inferior parietal in response scenes from online games, relative to control group at baseline. 2) Improvement in perceived family cohesion following 3 weeks of treatment was associated with an increase in the activity of the caudate nucleus in response to affection stimuli and was inversely correlated with changes in online game playing time.

*VBM* voxel-based morphometric

*PFC* prefrontal cortex

*DLPFC* dorsolateral prefrontal cortex

*OFC* orbitofrontal cortex

*ACC* anterior cingulate cortex

*SMA* supplementary motor area

*NAc* nucleus accumbens

*PCC* posterior cingulate cortex

*CBF* cerebral blood flow

*IAT* Internet Addiction Test

*YDQ* Young Diagnostic Questionnaire

*IGD* Internet Gaming Disorder

*OGA* Online Gaming Addiction

A study using the balloon analog risk task (BART), which evaluates the modulation of risk level (the probability of balloon explosion), reported that an IGD group showed reduced modulation of risk level regarding activation of the right DLPFC during the active BART and a negative correlation between risk-related DLPFC activation during the active BART and (self-rated) impulsivity [56].

A study using a working memory test showed greater activity in the right middle occipital gyrus, left cerebellar posterior lobe, left premotor cortex, and left middle temporal gyrus in response to working memory tasks during baseline measurements in adolescents with excessive online game playing [57]. The participants with excessive online game playing in the study showed increased activity within the right DLPFC and left occipital fusiform gyrus, and a negative correlation was found between changes in the severity of online game playing and changes in the mean beta value of the right DLPFC in response to a complex calculation task after 4 weeks of treatment.

A study using a task that required viewing scenes of affection and game scenes without responding reported decreased activity within the caudate nucleus, middle temporal gyrus, and occipital lobe in response to images depicting parental affection, and increased activity of the middle frontal and inferior parietal lobes in response to scenes from online games in adolescents with online gaming addiction relative to the control group at baseline [50].

A study using a task to determine the level of negative feelings induced by word stimuli indicated reduced activation in the right OFC, which was related to cognitive control, and in the dorsal ACC, which was related to social rejection, during the “swear word” condition, and a negative correlation between activity in the right amygdala during the “swear word” condition and anger control in adolescents with IGD [58].

Combined with the results of resting state brain imaging studies, abnormalities in the PFC and striatum regions may be associated with cognitive function relating to impulsivity in minors with IGD.

#### Effect of psychological intervention on brain imaging

A brief 3-week family therapy course for adolescents with online gaming addiction decreased activity, as measured by functional magnetic resonance imaging (fMRI), within the caudate nucleus, middle temporal gyrus, and occipital lobe in response to images depicting parental affection and increased activity of the middle frontal and inferior parietal lobes in response to scenes from online games. Improvement in family cohesion following the family therapy intervention was associated with an increase in the activity of the caudate nucleus in response to affection stimuli, and this was inversely correlated with changes in online game playing time [50].

#### Brain studies in adolescents with IGD and ADHD

Adolescents with IGD and ADHD, and those with ADHD alone were both characterized by decreased N-acetyl-aspartate levels within the frontal lobe, consistent with the hypofrontality characterized both in addiction and ADHD [59].

A study using quantitative electroencephalography reported that adolescents with IGD and ADHD show lower relative delta (0.5–3.5 Hz) power and greater relative beta (12.5–35.0 Hz) power in temporal regions compared with adolescents with ADHD alone [60]. The relative theta power in the frontal regions was significantly higher in the ADHD-only group but not in adolescents with both IGD and ADHD when compared to the healthy control group. This suggests that continuous game playing induces complex competitions and interactions between inter-hemispheric neurons.

#### Autonomic activity

A study of autonomic function in adolescents with IGD indicated lower heart rate variability parameters in adolescents with IGD compared to controls, and higher sympathetic and lower parasympathetic activity in the IGD group [61]. Additionally, the prevalence of a type D (distress) personality was nearly twice as common in the IGD group as in the non-IGD group, and type D personality negatively correlated with the logarithmic value of total power and low frequency among the heart rate variability parameters.

#### Genetic features

Taq1A1 of the dopamine D2 receptor and low activity catecholamine-O-methyltransferase (COMT) alleles were significantly more prevalent in adolescents with excessive internet video game play relative to a comparison group, and the presence of the Taq1A1 allele correlated with higher reward-dependence scores in adolescents with excessive internet video game play [62].

## Discussion

The results regarding the prevalence of IGD show slight differences depending on the method of classification used. Thus, the results of studies that used different questionnaires prior to the DSM-5 may be effectively referenced to studies that employed the DSM-5 for classification. Additionally, studies of participants including adults reported a prevalence of IGD of 0.5–27.5% [63, 64]; thus, variation in the prevalence of IGD appears to differ between adolescent samples and samples including adults. One possible reason for this is that the gaming time in minors could be controlled by environmental factors because school-age children may be prevented from online game play during their time in school. However, more important is that previous investigations of students did not include minors who stop attending school as participants. Thus, these investigations could have missed minors who start missing school owing to severe IGD. Future studies should focus on minors who were not included as participants in previous studies. Male adolescents are likely to have problematic online game use, consistent with the results of previous studies including adult participants. Mentzoni et al. [65] noted that 15.4% of male adolescents aged 16 to 21 years and 9.7% of young men aged 22 to 27 years had problematic video game use, while the rates in all other age and sex groups were under 3%. Regarding game genre, MMORPGs and FPSs were predominantly played by minors with IGD and strongly predicted the severity of IGD [5, 12]. Müller et al. [12] suggested that the characteristics of MMORPGs, including overstimulation of sensory and cognitive components of curiosity, social interactions, role-playing, and competition with like-minded individuals, could contribute to enhanced absorption in online game playing. The FPSs are “kill-or-be-killed” games from the player’s perspective, thus FPS players may be easily immersed in the game compared with third-person shooter games.

In minors, the presence of IGD interferes with daily living relating to schoolwork, including skipping school classes [14] and academic performance [12]. The severe sleep disturbances shown in minors with IGD [14] might be related to these interferences in school work. Further, students with poor school performance could be more likely to have IGD.

Family relationships were reported as an important social factor in minors with IGD. A systematic review reported that poor quality parent-child relationships are associated with increased severity of problem gaming, and that the paternal relationship may be protective against problem gaming [66]. Given the results of our review and the previous systematic review, parent-child relationships may be related to the presence or severity of IGD. Family therapy for adolescents with IGD was reported to be effective in improving IGD symptoms and brain activation changes in response to online game playing cues and images depicting parental love; one report concluded that family cohesion may be an important factor in the treatment of IGD in adolescents [50]. A study that surveyed players of MMORPGs aged 18–32 showed that almost half (47%) of the problem gamers reported features of disorganized attachment and playing to escape from painful memories of abuse [67]. Domestic abuse should be investigated as one of the risk factors for the development of IGD in children and adolescents. Regarding parental involvement in the development of IGD in children, our review indicated that parental attitudes that hinder or foster IGD could differ by the sex of the minors; rules about gaming use are important in male adolescents to prevent IGD, while for female adolescents a ban from gaming could prevent excessive gaming use [44]. A systematic review suggested that the support of cooperative fathers may play an important role in preventive programs because longitudinal evidence indicated that the paternal bond was a protective factor against problematic game use [66]. Thus, in addition to the sex of minors, family members may be important in the development of IGD, including both the mother and the father and others who intervene in children’s game use. Additionally, both the mental health of minors and their parents were reported to be associated with the presence of IGD [46]; thus, therapists should consider parents’ mental health in cases of minors with IGD.

Various psychological and social factors were found in minors with IGD. In particular, the problem of impulsiveness was reported as an important psychological factor in IGD. Young [4] defined Internet addiction, including IGD, as an impulse control disorder. Given the results of our review, impulsiveness was related to cognitive dysfunction found in brain imaging studies. A cognitive-behavioral model of IGD indicated that executive function promoting cognitive and behavioral control inhibits the craving for online game use, and that online gaming behavior disturbs executive function [68]. The executive function underlying impulsiveness may be targeted as an important component of psychological treatment of IGD, including in cognitive behavioral therapy and cognitive enhancement therapy. Sakuma et al. [51] demonstrated the effectiveness of a therapeutic residential camp that included cognitive behavioral therapy sessions for decreased gaming time and increased self-efficacy in adolescents with IGD. Additionally, 4-week virtual reality therapy displaying relaxing videos, gaming cues, and aversion-inducing stimuli for adults with online gaming addiction also reduced the severity of online gaming addiction, showing effects equivalent to those of cognitive behavior therapy, and enhanced balance of the cortico-striatal-limbic circuit [69]. The development of effective psychological interventions for minors with IGD is an urgent issue that requires further research. In a clinical setting, detecting a problem at an early stage and providing appropriate treatment has a marked influence on patient prognosis. In particular, the personality of adolescents is still immature. Before the brain matures, adequate interventions and actions must be performed to promote brain development. This is applicable not only to IGD but also to many psychiatric problems [70].

Park et al. [39] longitudinally demonstrated a correlation between IGD severity and impulsiveness as selected characteristics of ADHD in adolescents with ADHD and IGD. Previous studies reported high comorbidity of ADHD and internet addiction [71]. A self-rating survey reported that college students who screened positive for ADHD were more likely to be problematic internet users [72]. Minors with ADHD appear to be vulnerable to developing problematic video game use [73, 74]. A correlation between visual working memory and methylphenidate efficacy has been reported previously [75, 76]. Linking clinical symptoms and visual attention, Pasini et al. [77] suggested that impairments in visual-object working memory might be a neuropsychological trait of ADHD (inattentive or combined subtypes). A previous study using the Stroop task reported that individuals with IGD showed impairments in both visual and cognitive control ability while processing gaming-related words [78]. Thus, impaired cognitive function could mediate the relationship between IGD and ADHD.

A review of brain imaging studies in young adults with IGD indicated that the more relevant abnormalities were localized to the superior temporal gyrus, limbic system, and medial frontal and parietal regions in the resting state. Fewer than half of the task-related fMRI studies reported behavioral differences between patients and controls, but all such studies found significant differences in cortical and subcortical brain regions involved in cognitive control and reward processing: OFC, insula, ACC, posterior cingulate cortex, temporal and parietal regions, brain stem, and caudate nucleus [79]. Our review also indicated similar characteristics in minors. The PFC, including both the ventromedial and dorsolateral regions, is considered to play an important role in cognitive processes [80–86]. Brain imaging studies in individuals with IGD have supported the existence of a relationship between the PFC and impaired executive function, including basic cognitive processes such as cognitive inhibition, inhibitory control, or working memory. Ding et al. [34] suggested that the PFC may be involved in impulse control, and that impairments in its function may underlie the high rate of impulsivity in adolescents with IGD. Some studies including adults with IGD have indicated impaired cognitive control ability [87–91]. Additionally, Cai et al. [55] demonstrated an important role of the striatum, caudate nucleus, and NAc in cognitive control and the severity of IGD in adolescents. A study of individuals with IGD [92] including adults also indicated that the caudate volume was correlated with impaired cognitive control. The caudate nucleus has biological connections with the PFC, particularly the DLPFC, as a prefrontal-striatal circuit [87], and both the DLPFC and the prefrontal-striatal circuits play a crucial role in cognitive control [93]. The caudate nucleus is most frequently mentioned as an important target for dopamine in addiction since it plays a major role in conditioned reinforcement and reward expectation [94]. Reduced dopamine receptor availability in the dorsal caudate nucleus has been detected in individuals with IGD [95]. In late adolescents, including participants aged 20 or above, with online gaming addiction, Yuan et al. [90] reported increased thickness of the left precentral cortex, and cortical thickness of the left precentral cortex correlated with the duration of online gaming addiction. Taken together, cortical thickness changes may be associated with the process of acquiring better playing skills, namely transforming from a “rookie” to an “advanced player.” Persistent pathological online gaming since childhood could aggravate abnormal brain function, and preventive care and early intervention is increasingly important.

Genetic features and environmental factors, including family relationships, could increase the vulnerability to IGD in minors, and abnormal brain function could maintain cognitive, behavioral, emotional, and biological symptoms.

## Conclusion

We summarized biopsychosocial features in children and adolescents with IGD by reviewing relevant previous studies. The presence of IGD has a negative effect on sleep and schoolwork in minors. Family factors, including the quality of the parent-child relationship, are important social factor in minors with IGD, and both the mental health of minors and their parents may be associated with the presence of IGD. Given the results from adults, domestic abuse should be investigated as a risk factor for the development of IGD in minors. Additionally, many brain imaging studies in minors with IGD have indicated that their impaired cognitive control functions are associated with abnormal function in the PFC and striatum. Persistent pathological online game use from childhood could aggravate abnormal brain function. Preventive care and early intervention for IGD in minors is important. Although the effectiveness of cognitive behavioral therapy for minors with IGD has been supported, effective psychological intervention for minors with IGD is an urgent issue that requires further research.
